# Cyanoacrylate glue prevents early bleeding of the exit site after CVC or PICC placement

**DOI:** 10.1186/cc12112

**Published:** 2013-03-19

**Authors:** G Scoppettuolo, MG Annetta, C Marano, E Tanzarella, M Pittiruti

**Affiliations:** 1 Catholic University, Rome, Italy

## Introduction

Early bleeding from the exit site after CVC or PICC placement is a very common event that causes difficulties in the patient's care and logistical problems. In our experience, the rate of significant local bleeding after placement of PICCs without reverse tapering may be as high as 40% at 1 hour and 15% at 24 hours, while the rate of bleeding after placement of a large-bore dialysis catheter is above 50% at 1 hour.

## Methods

The aim of this pilot study was to verify the efficacy of a cyanoacrylate glue in reducing the risk of early bleeding at the exit site after CVC or PICC placement. We studied a group of adult patients consecutively undergoing placement of polyurethane CVCs or PICCs without reverse tapering in a non-intensive ward of our hospital. All lines were inserted according to the same protocol, which included 2% chlorhexidine antisepsis, maximal sterile barriers, ultrasound guidance, EKG guidance and securement with sutureless device. Two minutes after placement of the glue, the exit site was covered with a temporary gauze dressing, which was replaced by transparent membrane at 24 hours. All patients were assessed at 1 hour and at 24 hours.

## Results

In 65 consecutive patients (45 PICCs, 11 dialysis catheters and nine CVCs), there was no significant local bleeding at 1 hour or at 24 hours after catheter placement. No local adverse reaction occurred. No damage to the polyurethane of the catheters was detected.

## Conclusion

Glue is an inexpensive and highly effective tool for avoiding the risk of early bleeding of the exit site after catheter placement. We also suggest that in the next future the glue might prove to have beneficial collateral effects on the risk of extraluminal contamination (by reducing the entrance of bacteria in the space between the catheter and the skin), as well as on the risk of dislocation (by increasing the stability of the catheter inside the skin breach).

